# Supplementary material for: Rapid Classification and Quantification of Camellia (Camellia oleifera Abel.) Oil Blended with Rapeseed Oil Using FTIR-ATR Spectroscopy
Source: Molecules. 2020 Apr 27;25(9):2036. doi: 10.3390/molecules25092036 (PMC7248856; doi:10.3390/molecules25092036)
Supplement: Supplementary file 1 [file molecules-25-02036-s001.pdf]

SUPPLEMENTARY MATERIALS

Supplementary figures

Supplementary Figure S1. The average FTIR spectra with error bars of CAO, SBO, CO, RSO, PO, SFO, and SO in the full region of 4000–650 cm^{-1}. CAO, Camellia oil; SBO, Soybean oil; RSO, Rapeseed oil; CO, Corn oil; SFO, Sunflower oil; PO, Peanut oil; SO, Sesame oil.

Supplementary Figure S2. Correlation of I_{1119}/I_{1096} ratios with the concentration of RSO blended with CAO. CAO, Camellia oil; RSO, Rapeseed oil.

Supplementary Figure S3. Partial least squares regression (PLSR) calibration model in the regions of 1800 cm^{-1}-650 cm^{-1} for relationship between actual (Reference Y) versus FTIR predicted adulteration level of RSO-1 in CAO-1 (Predicted Y).

Supplementary Figure S4. Partial least squares regression (PLSR) calibration model in the regions of 1800 cm^{-1}-650 cm^{-1} for relationship between actual (Reference Y) versus FTIR predicted adulteration level of RSO-1 in CAO-2 (Predicted Y).

Supplementary Figure S5. Partial least squares regression (PLSR) calibration model in the regions of 1800 cm^{-1}-650 cm^{-1} for relationship between actual (Reference Y) versus FTIR predicted adulteration level of RSO-1 in CAO-3 (Predicted Y).

Supplementary Figure S6. Partial least squares regression (PLSR) calibration model in the regions of 1800 cm^{-1}-650 cm^{-1} for relationship between actual (Reference Y) versus FTIR predicted adulteration level of RSO-1 in CAO-4 (Predicted Y).

Supplementary Figure S7. Partial least squares regression (PLSR) calibration model in the regions of 1800 cm^{-1}-650 cm^{-1} for relationship between actual (Reference Y) versus FTIR predicted adulteration level of RSO-1 in CAO-5 (Predicted Y).

Supplementary Figure S8. Partial least squares regression (PLSR) calibration model in the regions of 1800 cm^{-1}-650 cm^{-1} for relationship between actual (Reference Y) versus FTIR predicted adulteration level of RSO-2 in CAO-1 (Predicted Y).

Supplementary Figure S9. Partial least squares regression (PLSR) calibration model in the regions of 1800 cm^{-1}-650 cm^{-1} for relationship between actual (Reference Y) versus FTIR predicted adulteration level of RSO-2 in CAO-2 (Predicted Y).

Supplementary Figure S10. Partial least squares regression (PLSR) calibration model in the regions of 1800 cm^{-1}-650 cm^{-1} for relationship between actual (Reference Y) versus FTIR predicted adulteration level of RSO-2 in CAO-3 (Predicted Y).

Supplementary Figure S11. Partial least squares regression (PLSR) calibration model in the regions of 1800 cm^{-1}-650 cm^{-1} for relationship between actual (Reference Y) versus FTIR predicted adulteration level of RSO-2 in CAO-4 (Predicted Y).

Supplementary Figure S12. Partial least squares regression (PLSR) calibration model in the regions of 1800 cm^{-1}-650 cm^{-1} for relationship between actual

(Reference Y) versus FTIR predicted adulteration level of RSO-2 in CAO-5 (Predicted Y).

Supplementary Figure S13. Partial least squares regression (PLSR) calibration model in the regions of 1800 cm^{-1}-650 cm^{-1} for relationship between actual (Reference Y) versus FTIR predicted adulteration level of RSO-3 in CAO-1 (Predicted Y).

Supplementary Figure S14. Partial least squares regression (PLSR) calibration model in the regions of 1800 cm^{-1}-650 cm^{-1} for relationship between actual (Reference Y) versus FTIR predicted adulteration level of RSO-3 in CAO-2 (Predicted Y).

Supplementary Figure S15. Partial least squares regression (PLSR) calibration model in the regions of 1800 cm^{-1}-650 cm^{-1} for relationship between actual (Reference Y) versus FTIR predicted adulteration level of RSO-3 in CAO-3 (Predicted Y).

Supplementary Figure S16. Partial least squares regression (PLSR) calibration model in the regions of 1800 cm^{-1}-650 cm^{-1} for relationship between actual (Reference Y) versus FTIR predicted adulteration level of RSO-3 in CAO-4 (Predicted Y).

Supplementary Figure S17. Partial least squares regression (PLSR) calibration model in the regions of 1800 cm^{-1}-650 cm^{-1} for relationship between actual (Reference Y) versus FTIR predicted adulteration level of RSO-3 in CAO-5 (Predicted Y).

Supplementary tables

**Supplementary Table S1.**	Relationship between I_{1119}/I_{1096} intensity ratio and concentration of adulterated RSO oil in the binary mixtures.
**Supplementary Table S2.**	Confusion matrix data table of LDA model for RSO 10%, RSO 5%, RSO 3%, RSO 1% V/V, and pure CAO.
**Supplementary Table S3.**	Classification table of LDA model for studied samples.
**Supplementary Table S4.**	Detailed information of edible vegetable oils.

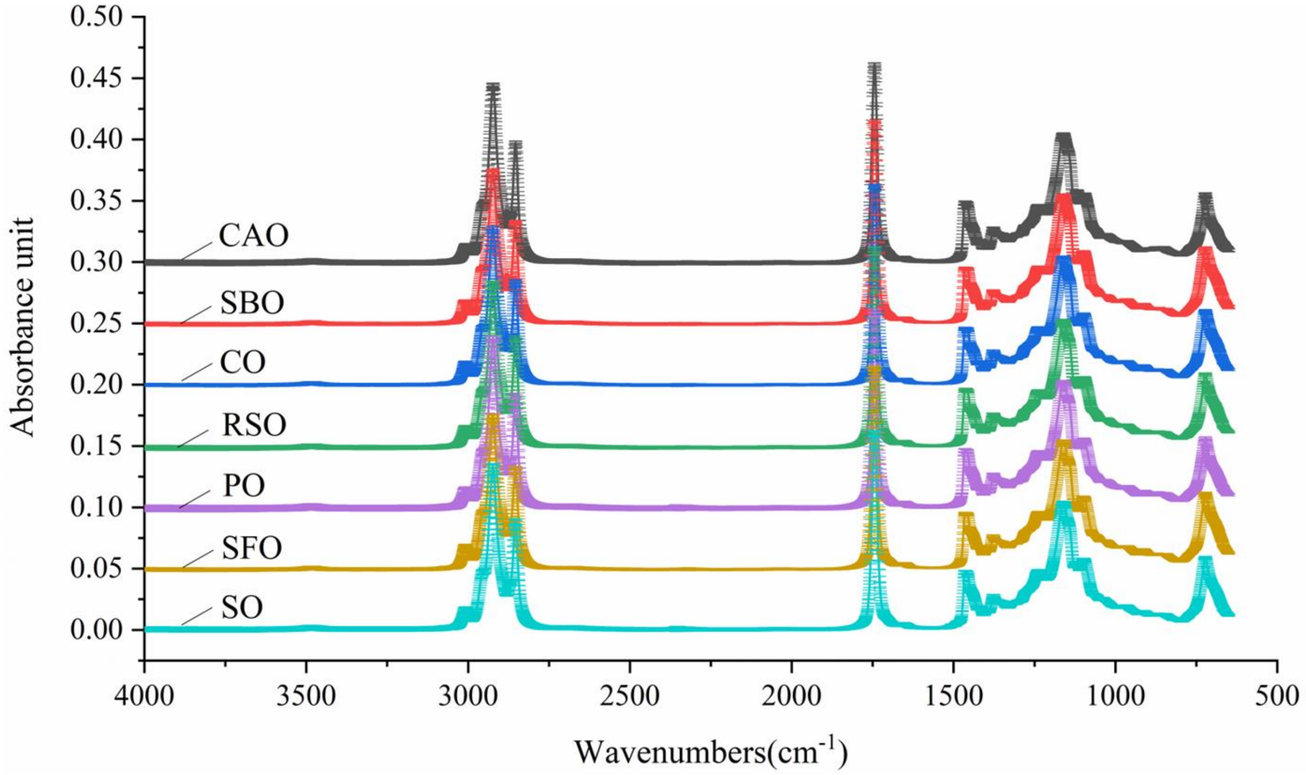

Supplementary Figure S1

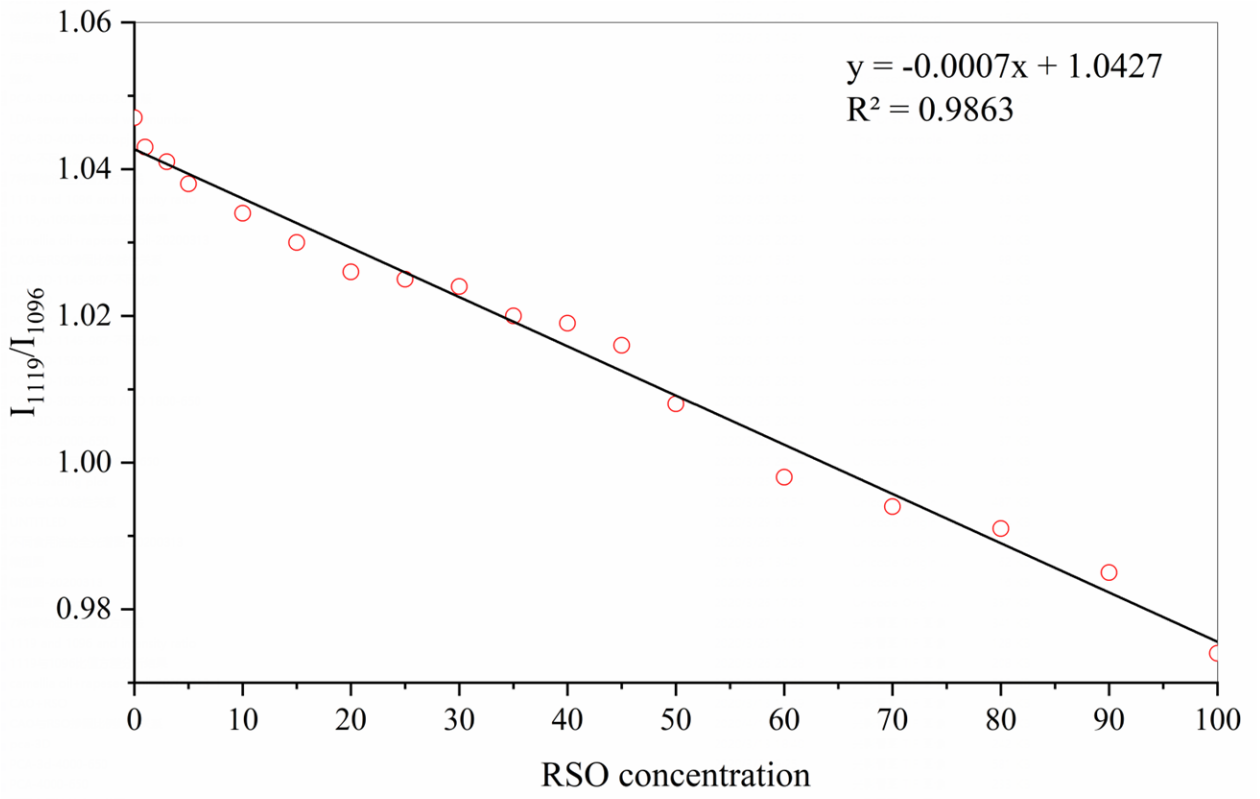

Supplementary Figure S2

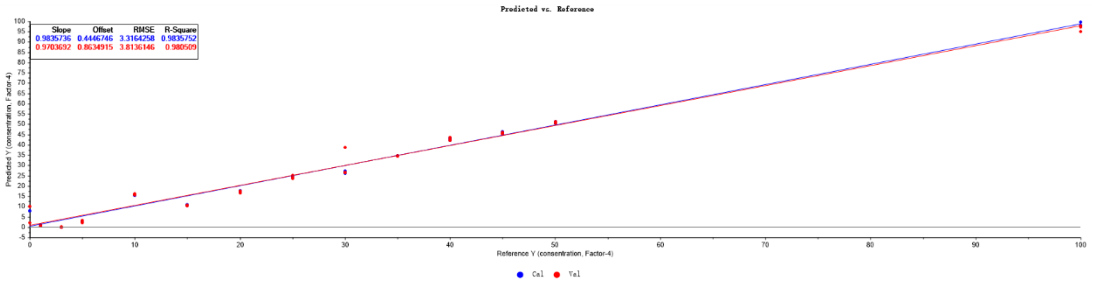

Supplementary Figure S3

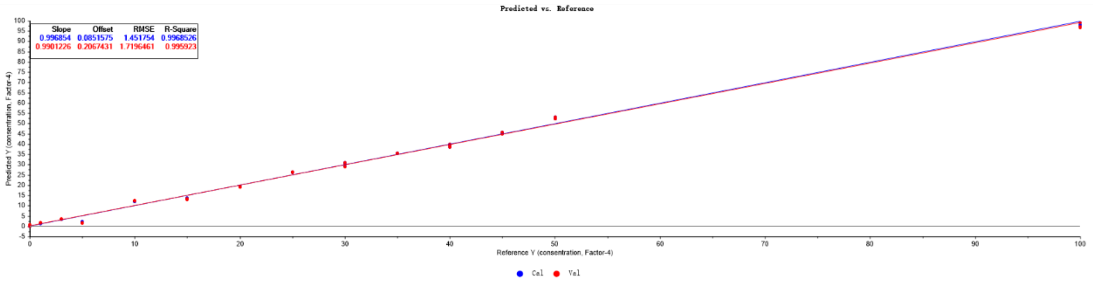

Supplementary Figure S4

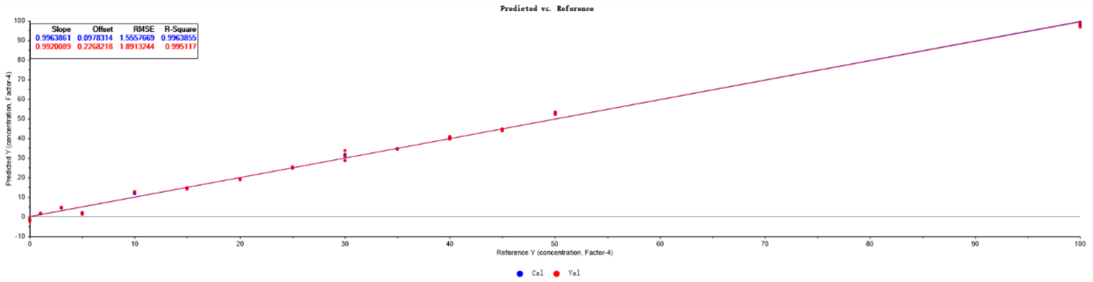

Supplementary Figure S5

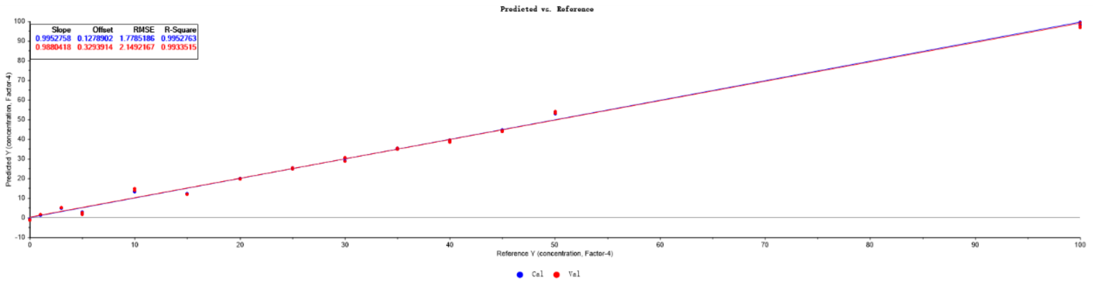

Supplementary Figure S6

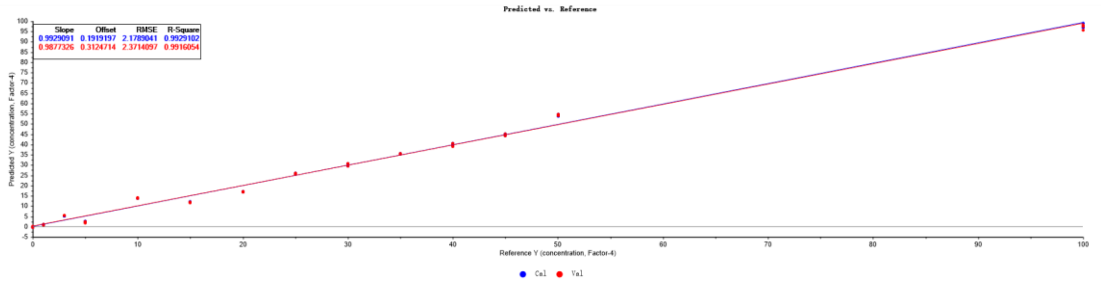

Supplementary Figure S7

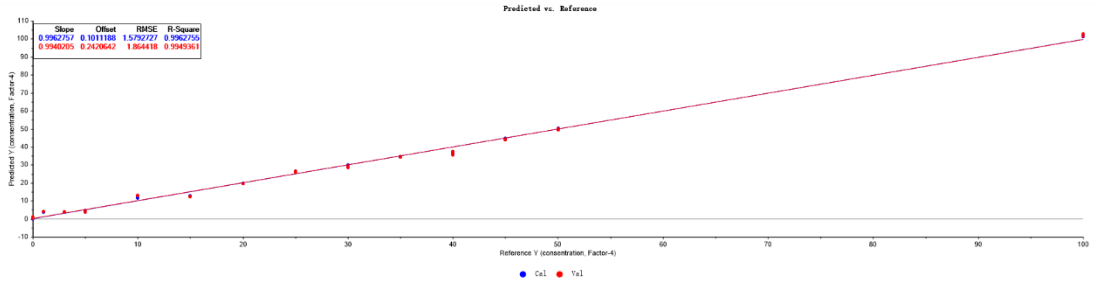

Supplementary Figure S8

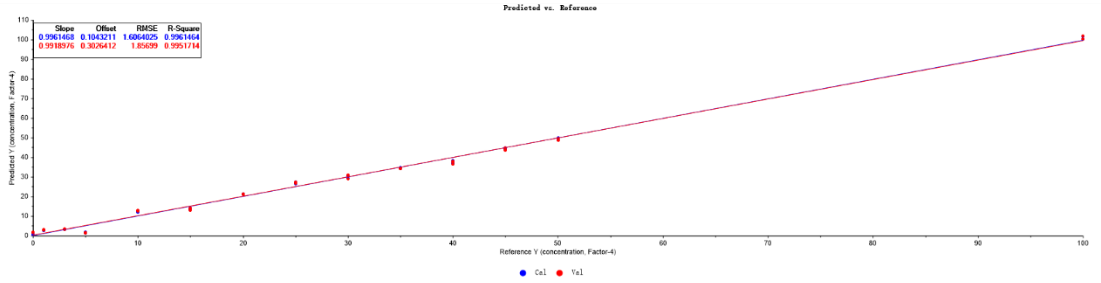

Supplementary Figure S9

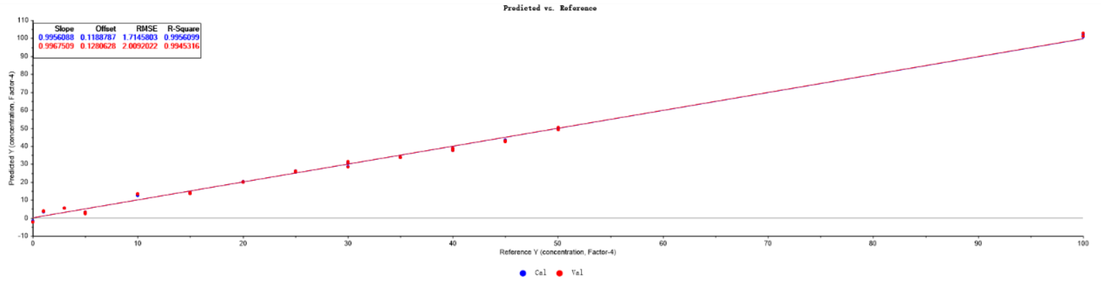

Supplementary Figure S10

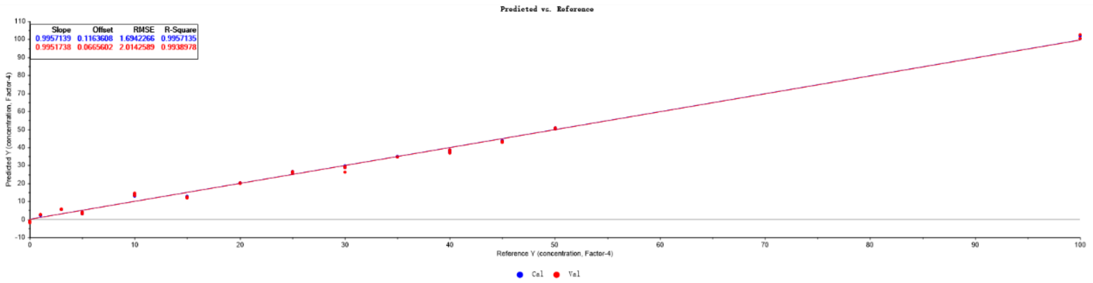

Supplementary Figure S11

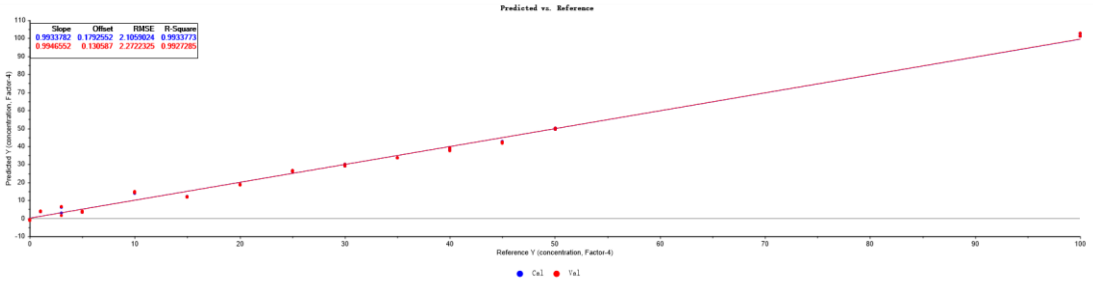

Supplementary Figure S12

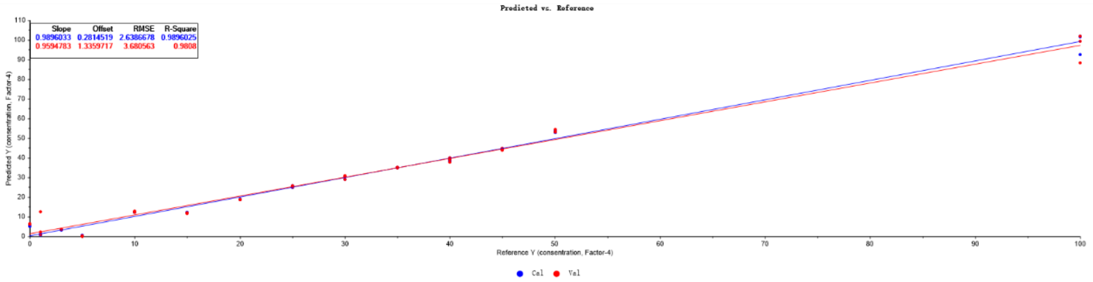

Supplementary Figure S13

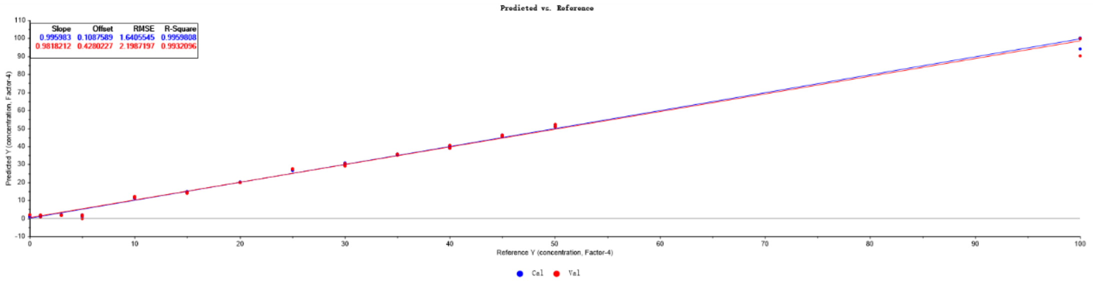

Supplementary Figure S14

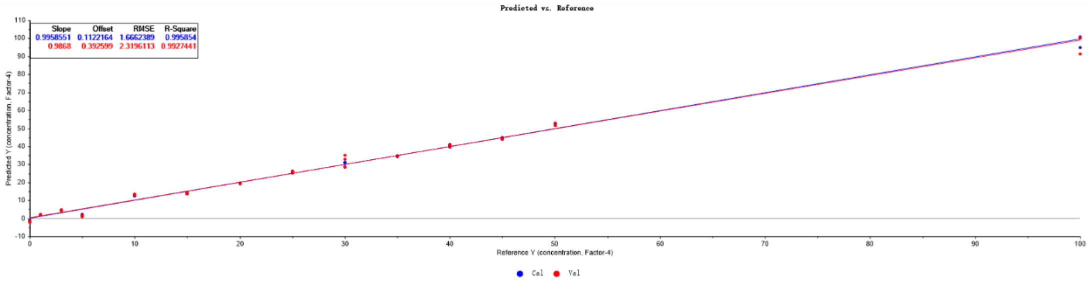

Supplementary Figure S15

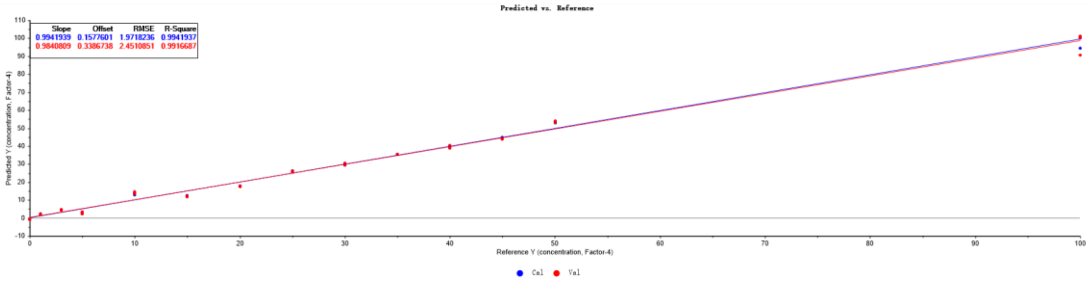

Supplementary Figure S16

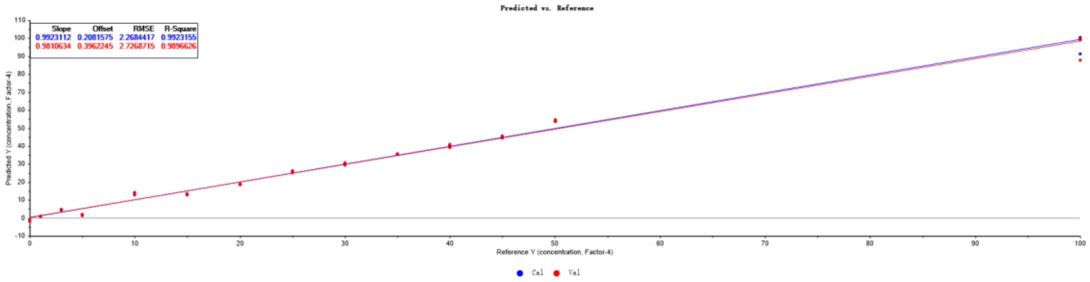

Supplementary Figure S17

Supplementary Table S1

Concentration of RSO in the CAO/RSO admixtures (%)	I_{1119}/I_{1096} of CAO/RSO admixtures[a]
0	1.047± 0.001
1	1.043 ± 0.001
3	1.041 ± 0.002
5	1.038 ± 0.000
10	1.034 ± 0.002
15	1.030 ± 0.000
20	1.026 ± 0.001
25	1.025 ± 0.000
30	1.024 ± 0.000
35	1.020 ± 0.001
40	1.019 ± 0.001
45	1.016 ± 0.001
50	1.008 ± 0.000
60	0.998 ± 0.001
70	0.994 ± 0.001
80	0.991 ± 0.001
90	0.985 ± 0.001
100	0.974 ± 0.001

[a]Each value is a mean ± standard deviation of triplicate determinations.

Supplementary Table S2

Confusion matrix	Actual	CAO	RSO 1%	RSO 3%	RSO 5%	RSO 10%
Predicted		1	2	3	4	5
CAO	1	15	0	0	0	0
RSO 1%	2	0	15	0	0	0
RSO 3%	3	0	0	15	0	0
RSO 5%	4	0	0	0	15	0
RSO 10%	5	0	0	0	0	15

Supplementary Table S3

Classified_LDA-test-1_Sheet1		CAO	RSO 1%	RSO 3%	RSO 5%	RSO 10%	Class
		1	2	3	4	5	6
CAO#1	1	-4.1129	-1366.0530	-1627.2960	-2275.4680	-3434.4690	CAO
CAO#2	2	-4.1129	-1366.0530	-1627.2960	-2275.4680	-3434.4690	CAO
CAO#3	3	-4.1129	-1366.0530	-1627.2960	-2275.4680	-3434.4690	CAO
CAO#4	4	-5.7691	-1366.6660	-1636.3650	-2298.7190	-3476.8010	CAO
CAO#5	5	-5.7691	-1366.6660	-1636.3650	-2298.7190	-3476.8010	CAO
CAO#6	6	-5.7691	-1366.6660	-1636.3650	-2298.7190	-3476.8010	CAO
CAO#7	7	-6.8982	-1296.4230	-1559.6680	-2195.1640	-3318.6520	CAO
CAO#8	8	-6.8982	-1296.4230	-1559.6680	-2195.1640	-3318.6520	CAO
CAO#9	9	-6.8982	-1296.4230	-1559.6680	-2195.1640	-3318.6520	CAO
CAO#10	10	-7.4697	-1416.9280	-1679.7620	-2357.4120	-3541.7630	CAO
CAO#11	11	-7.4697	-1416.9280	-1679.7620	-2357.4120	-3541.7630	CAO
CAO#12	12	-7.4697	-1416.9280	-1679.7620	-2357.4120	-3541.7630	CAO
CAO#13	13	-5.0382	-1264.5630	-1516.4320	-2140.5020	-3273.2500	CAO
CAO#14	14	-4.7332	-1322.3000	-1580.5640	-2242.6810	-3381.4780	CAO
CAO#15	15	-4.7332	-1322.3000	-1580.5640	-2242.6810	-3381.4780	CAO
RSO 1%#1	16	-1437.0500	-5.8988	-24.9165	-207.5531	-552.7589	RSO 1%
RSO 1%#2	17	-1437.0500	-5.8988	-24.9165	-207.5531	-552.7589	RSO 1%
RSO 1%#3	18	-1390.1130	-6.4142	-27.1909	-207.0557	-576.5154	RSO 1%
RSO 1%#4	19	-1390.1130	-6.4142	-27.1909	-207.0557	-576.5154	RSO 1%
RSO 1%#5	20	-1486.6100	-8.0070	-25.2656	-210.6729	-531.6240	RSO 1%
RSO 1%#6	21	-1317.8000	-6.2447	-53.4464	-263.1779	-631.1279	RSO 1%
RSO 1%#7	22	-1354.0080	-10.6384	-59.4512	-286.0870	-624.7234	RSO 1%
RSO 1%#8	23	-1287.9510	-8.2330	-53.8267	-246.6584	-643.9281	RSO 1%
RSO 1%#9	24	-1296.9180	-2.4667	-47.9582	-255.8349	-629.8547	RSO 1%
RSO 1%#10	25	-1287.9510	-8.2330	-53.8267	-246.6584	-643.9281	RSO 1%
RSO 1%#11	26	-1401.3970	-7.4204	-51.2253	-247.8812	-567.3127	RSO 1%
RSO 1%#12	27	-1302.8870	-3.8155	-51.1383	-242.2522	-610.1931	RSO 1%
RSO 1%#13	28	-1257.5780	-5.9483	-55.0292	-243.3693	-635.5628	RSO 1%
RSO 1%#14	29	-1257.5780	-5.9483	-55.0292	-243.3693	-635.5628	RSO 1%
RSO 1%#15	30	-1350.8300	-4.3059	-49.8698	-243.7553	-587.4423	RSO 1%
RSO 3%#1	31	-1570.5600	-32.4763	-2.2303	-132.2826	-453.5087	RSO 3%
RSO 3%#2	32	-1648.7930	-35.9341	-3.9223	-126.7446	-431.8625	RSO 3%
RSO 3%#3	33	-1591.7130	-54.5080	-4.3273	-122.2539	-438.5102	RSO 3%
RSO 3%#4	34	-1595.0990	-33.2871	-2.1614	-133.5988	-442.6974	RSO 3%
RSO 3%#5	35	-1593.2490	-40.0550	-6.7885	-122.9220	-424.8851	RSO 3%
RSO 3%#6	36	-1613.7070	-36.0915	-7.4846	-155.5610	-448.5391	RSO 3%
RSO 3%#7	37	-1546.6790	-32.3217	-2.9550	-131.6213	-464.9746	RSO 3%
RSO 3%#8	38	-1624.5870	-35.4564	-4.3241	-125.7612	-443.0065	RSO 3%
RSO 3%#9	39	-1674.5520	-36.4300	-4.1493	-122.7710	-413.7364	RSO 3%
RSO 3%#10	40	-1673.6620	-37.0684	-4.1762	-128.3819	-421.3699	RSO 3%
RSO 3%#11	41	-1648.7930	-35.9341	-3.9223	-126.7446	-431.8625	RSO 3%
RSO 3%#12	42	-1591.7130	-54.5080	-4.3273	-122.2539	-438.5102	RSO 3%
RSO 3%#13	43	-1596.5980	-54.6437	-4.2864	-122.4886	-436.3173	RSO 3%
RSO 3%#14	44	-1596.5980	-54.6437	-4.2864	-122.4886	-436.3173	RSO 3%
RSO 3%#15	45	-1596.5980	-54.6437	-4.2864	-122.4886	-436.3173	RSO 3%
RSO 5%#1	46	-2161.3040	-213.7110	-111.8066	-3.3420	-205.3815	RSO 5%
RSO 5%#2	47	-2273.1070	-246.8162	-136.4163	-2.4636	-190.3749	RSO 5%
RSO 5%#3	48	-2245.0810	-222.7294	-119.0604	-3.3692	-189.3043	RSO 5%
RSO 5%#4	49	-2184.0830	-212.7847	-110.0029	-2.9285	-192.8461	RSO 5%
RSO 5%#5	50	-2246.5430	-222.9367	-113.7750	-5.3892	-179.0929	RSO 5%
RSO 5%#6	51	-2255.7690	-229.7407	-118.2695	-7.8436	-185.5691	RSO 5%
RSO 5%#7	52	-2165.8160	-213.4760	-111.3953	-3.2066	-202.8203	RSO 5%
RSO 5%#8	53	-2277.6780	-246.6464	-136.0708	-2.3959	-187.8825	RSO 5%
RSO 5%#9	54	-2506.6570	-326.0980	-199.3809	-17.2783	-175.9209	RSO 5%
RSO 5%#10	55	-2262.2920	-238.0336	-133.9243	-5.9589	-180.1817	RSO 5%
RSO 5%#11	56	-2262.2920	-238.0336	-133.9243	-5.9589	-180.1817	RSO 5%
RSO 5%#12	57	-2234.3370	-214.0054	-116.6260	-6.9200	-179.1639	RSO 5%
RSO 5%#13	58	-2294.2500	-235.6647	-128.4206	-2.8096	-180.6656	RSO 5%
RSO 5%#14	59	-2268.1980	-222.1283	-117.5810	-3.2781	-177.0885	RSO 5%
RSO 5%#15	60	-2358.2610	-257.2116	-145.0471	-3.8679	-175.6746	RSO 5%
RSO 10%#1	61	-3368.8050	-572.4855	-420.9579	-165.5514	-4.3050	RSO 10%
RSO 10%#2	62	-3349.8970	-568.3561	-412.2462	-163.8858	-3.6082	RSO 10%
RSO 10%#3	63	-3476.3230	-653.2498	-489.2942	-202.7960	-7.0632	RSO 10%
RSO 10%#4	64	-3403.1470	-583.1044	-430.6982	-176.6780	-3.3054	RSO 10%
RSO 10%#5	65	-3480.5800	-608.2418	-449.4565	-194.1271	-4.5431	RSO 10%
RSO 10%#6	66	-3480.5800	-608.2418	-449.4565	-194.1271	-4.5431	RSO 10%
RSO 10%#7	67	-3480.5800	-608.2418	-449.4565	-194.1271	-4.5431	RSO 10%
RSO 10%#8	68	-3463.0640	-605.5096	-442.1424	-193.8610	-5.2474	RSO 10%
RSO 10%#9	69	-3308.6370	-550.5396	-399.4830	-172.3186	-4.4479	RSO 10%
RSO 10%#10	70	-3386.5470	-572.5037	-413.8542	-178.7484	-4.3325	RSO 10%
RSO 10%#11	71	-3304.1330	-531.8441	-372.0724	-157.2397	-9.1209	RSO 10%
RSO 10%#12	72	-3423.8220	-610.0010	-442.3836	-189.4130	-5.8392	RSO 10%
RSO 10%#13	73	-3429.3680	-610.7083	-438.4720	-181.4230	-5.8945	RSO 10%
RSO 10%#14	74	-3492.5920	-648.8707	-482.8140	-208.8760	-5.0677	RSO 10%
RSO 10%#15	75	-3492.5920	-648.8707	-482.8140	-208.8760	-5.0677	RSO 10%

Supplementary Table S4

Vegetable oils	Number	Brand	Geographic Origin
Camellia oil	CAO-1	Jiaxiangle	Jiangxi, China
	CAO-2	Huangpaoshan	Hubei, China
	CAO-3	Changfa	Zhejiang, China
	CAO-4	Younibao	Guangdong, China
	CAO-5	Shanggutang	Guangxi, China
	CAO-6	Shanghangjinse	Fujian, China
	CAO-7	Huayin	Anhui, China
Rapeseed oil	RSO-1	Fulinmen	Jiangsu, China
	RSO-2	Caiziwang	Jiangsu, China
	RSO-3	Jinlongyu	Jiangsu, China
	RSO-4	Yangyanghong	Jiangsu, China
	RSO-5	Luhua	Jiangsu, China
	RSO-6	Weizeyuan	Chongqing, China
	RSO-7	Liyu	Sichuan, China
Soyabean oil	SBO-1	Fulinmen	Jiangsu, China
	SBO-2	Jinlongyu	Jiangsu, China
	SBO-3	Xiangmanyuan	Jiangsu, China
	SBO-4	Fukangyuan	Jiangsu, China
	SBO-5	Luhua	Jiangsu, China
	SBO-6	Hongqingting	Chongqing, China
	SBO-7	Yuanbao	Chongqing, China
Sunflower oil	SFO-1	Fulinmen	Jiangsu, China
	SFO-2	Duoli	Jiangsu, China
	SFO-3	Luhua	Jiangsu, China
	SFO-4	Jinding	Jiangsu, China
	SFO-5	Jinlongyu	Zhejiang, China
	SFO-6	Xiwang	Shandong, China
	SFO-7	Duolihuangjinyi	Jiangsu, China
Corn oil	CO-1	Fulinmen	Jiangsu, China
	CO-2	Changshouhua	Shandong, China
	CO-3	Xiwang	Shandong, China
	CO-4	Luhua	Shandong, China
	CO-5	Jinlongyu	Jiangsu, China
	CO-6	Wufeng	Jiangsu, China
	CO-7	Duoli	Jiangsu, China
Peanut oil	PO-1	Jinlongyu	Jiangsu, China
	PO-2	Luhua	Jiangsu, China
	PO-3	Xiwang	Shandong, China
	PO-4	Hujihua	Shandong, China
	PO-5	Duoli	Jiangsu, China
	PO-6	Jinsheng	Shandong, China
	PO-7	Lianglong	Shanghai, China
Sesame oil	SO-1	Toudao	Jiangsu, China
	SO-2	Yanzhuang	Anhui, China
	SO-3	Runzhijia	Anhui, China

	SO-4	Xiangmanyuan	Hubei, China
	SO-5	Changkang	Hunan, China
	SO-6	Hongqingting	Chongqing, China
	SO-7	Luhua	Shandong, China
